# Event-related brain potentials to emotional images and gonadal steroid hormone levels in patients with schizophrenia and paired controls

**DOI:** 10.3389/fpsyg.2014.00543

**Published:** 2014-06-11

**Authors:** Julie Champagne, Adrianna Mendrek, Martine Germain, Pascal Hot, Marc E. Lavoie

**Affiliations:** ^1^Axe de Neurobiologie Cognitive, Laboratoire de Psychophysiologie Cognitive et Sociale, Centre de Recherche de l’Institut Universitaire en Santé Mentale de MontréalMontréal, QC, Canada; ^2^Department of Psychiatry, Université de MontréalMontréal, QC, Canada; ^3^Department of Psychology, Bishop’s University, SherbrookeQC, Canada; ^4^Laboratoire de Psychologie et Neurocognition, Université de SavoieChambéry, France

**Keywords:** schizophrenia, event related potential, emotions, gonadic hormones and sex differences

## Abstract

Prominent disturbances in the experience, expression, and emotion recognition in patients with schizophrenia have been relatively well documented over the last few years. Furthermore, sex differences in behavior and brain activity, associated with the processing of various emotions, have been reported in the general population and in schizophrenia patients. Others proposed that sex differences should be rather attributed to testosterone, which may play a role in the etiology of schizophrenia. Also, it had been suggested that estradiol may play a protective role in schizophrenia. Surprisingly, few studies investigating this pathology have focused on both brain substrates and gonadal steroid hormone levels, in emotional processing. In the present study, we investigated electrocortical responses related to emotional valence and arousal as well as gonadal steroid hormone levels in patients with schizophrenia. Event-Related Potentials (ERP) were recorded during exposition to emotional pictures in 18 patients with schizophrenia and in 24 control participants paired on intelligence, manual dominance and socioeconomic status. Given their previous sensitivity to emotional and attention processes, the P200, N200 and the P300 were selected for analysis. More precisely, emotional valence generally affects early components (N200), which reflect early process of selective attention, whereas emotional arousal and valence both influences the P300 component, which is related to memory context updating, and stimulus categorization. Results showed that, in the control group, the amplitude of the N200 was significantly more lateralized over the right hemisphere, while there was no such lateralization in patients with schizophrenia. In patients with schizophrenia, significantly smaller anterior P300 amplitude was observed to the unpleasant, compared to the pleasant. That anterior P300 reduction was also correlated with negative symptoms. The N200 and P300 amplitudes were positively correlated with the estradiol level in all conditions, revealing that the N200 and the P300 were reduced, when estradiol level was higher. Conversely, only the P300 amplitude showed positive correlation with the testosterone level.

## INTRODUCTION

Alteration of emotional functions in people with schizophrenia has been known for more than a century, back to the time of Kraepelin (Moskowitz and Heim, 2011) and Bleuler (Stotz-Ingenlath, 2000). At the end of the 1990s, several investigations have shown that the perception of emotions was impaired in patients with a diagnosis of schizophrenia, as well as in several other clinical groups, such as mood or bipolar disorders (Gaebel and Wolwer, 1992; Addington and Addington, 1998). A higher vulnerability to schizophrenia was also reported to be subtly manifested in altered emotional behavior in children, long before the onset of clinical symptoms (Walker et al., 1993). It is now well established that schizophrenia is associated with peculiarities in dealing with emotions, including an alteration of emotional expressiveness (Blanchard and Cohen, 2006), more prominent negative emotional traits (Horan et al., 2008), disorders of emotional signals perception (Hoekert et al., 2007), stronger unpleasant emotional experience (Cohen and Minor, 2010) and impaired capacity to infer other people emotional states (Koelkebeck et al., 2010). However, the neurocognitive roots of this emotional distinctiveness, present in schizophrenia, were only scrutinized recently with modern neuroimaging techniques. A meta-analysis by Taylor et al. (2012) has highlighted significant emotional differences in individuals affected by schizophrenia. During an emotional experience, patients with schizophrenia demonstrated greater bilateral reduced amygdala activation, as well as a reduced activation of the anterior cingulate cortex, of dorsolateral prefrontal cortex, the median frontal cortex and an increased activation of the cuneus, the parietal lobe, the precentral gyrus and the superior temporal gyrus.

As in schizophrenic patients, it is common to find a reduced global activation in response to the unpleasant valence images, compared to neutral ones, during basic emotional tasks *per se* and during emotional memory tasks as well (Schneider et al., 1998; Takahashi et al., 2004; Lakis et al., 2011a). For instance, Lakis et al. (2011b) showed that during a task of unpleasant image recognition, the cerebral activation was greater in the temporal and middle temporal gyrus, the precuneus, the cerebellum, the prefrontal cortex (orbitofrontal, upper and middle) and the cingulate gyrus in control individuals, while in schizophrenia patients exhibited significant activations only in the middle temporal cortex. Also, in individuals suffering from schizophrenia, it is common to denote an increase of activity in the frontal gyrus, the precuneus and median cingulate gyrus in response to neutral stimuli, compared with normal controls (Lakis et al., 2011b). This is consistent with the hypothesis that affective psychosis patients mobilize the limbic system to dedicate more importance to stimuli and events that should normally be regarded as neutral (Holt et al., 2006; Hall et al., 2008).

From these results, it is reasonable to infer that brain imaging is appropriate for identifying various localized metabolic variations in schizophrenia. However, it is limited by its low temporal resolution that does not fully take into account the real-time dynamic of the emotional systems (Logothetis, 2008). Event-related potentials (ERPs), provide robust clues to milliseconds accuracy cerebral activation and a plethora of research has already demonstrated its validity with emotional stimuli (Olofsson et al., 2008). Interestingly, a recent ERP study showed that emotionally evocative stimuli are differentially processed in schizophrenia patients within the first 200 ms, and that the early stages of emotional stimuli processing are particularly affected in that group (Pinheiro et al., 2013). Furthermore, another recent study underlined differences in patients with schizophrenia in later components, showing a lack of difference in the late positive component (LPC) in response to unpleasant images that followed neutral descriptors (Strauss et al., 2013). This could suggest a possible inability to downregulate emotional response.

In the last decade, several investigations have documented sex differences in emotion expression as well as emotional perception in schizophrenia. In general, men with schizophrenia tend to be more introverted and demonstrate a blunted affect more often, while women with schizophrenia show more irritability (Leung and Chue, 2000). Notwithstanding, among the works that support emotion processing specificities in individuals with schizophrenia, very few studies addressed simultaneously the questions of emotions, sex differences and hormonal fluctuations. Indeed, in comparing men and women’s brain activity, a significant amount of variance are explained by hormonal variations. For instance, testosterone and estradiol variations are correlated with cerebral activations during a mental rotation task (Mendrek et al., 2011). Moreover, during the exposition to a sequence of emotional images, progesterone level was positively correlated with cerebral activations (Champagne et al., 2012). But up till now, the effect of other gonadal steroid hormone variations, such as testosterone and estradiol, were not investigated in an ERP protocol with schizophrenia patients during exposition to emotional images.

The main goal of the current study was, thus, to assess processing of emotions in schizophrenia patients and the mediating influence of gonadal hormones on electrocortical activity. To achieve this goal, we exploited the temporal resolution of ERP in three time windows (N200, P200, and P300) to probe differences in schizophrenia. We explored these topics, while controlling for emotional arousal and pleasantness of standardized images in an orthogonal design. Few studies have systematically investigated arousal and valence as independent factors. According to the International Affective Picture System (Lang et al., 2008), valence is defined on a continuum of pleasantness, from the smallest value (1) as very unpleasant, and the highest value (9) as very unpleasant, while arousal represent the intensity of the emotion varying from a low value (1) to a highly arousing value (9).

It had been suggested that estradiol may play a protective role in schizophrenia (Hafner et al., 1993), while others proposed that sex differences should be attributed to testosterone, which may play a role in aggravating the etiology of schizophrenia (Beratis et al., 1994; Salokangas, 1995). In the current study, we propose that both estrogen and testosterone might play a role of modulator in emotional processing in these groups. Thus, we hypothesize that the insertion of gonadal hormones as covariates should statistically translate into the reduction (or the elimination) of the interactions (group by valence or group by arousal).

## MATERIALS AND METHODS

### PARTICIPANTS

The **Table 1** shows a socio-demographic and clinical description our groups. Eighteen patients with schizophrenia (9 men and 9 women) who met the diagnostic criteria of the DSM-IV for schizophrenia (APA, 1994) and were in a stable phase of their illness, and 24 control participants (11 men and 13 women) participated in the study. The two groups were matched for age, IQ, manual dominance (right-handed or left-handed) according to the test of Edinburg (Oldfield, 1971), and socio-economic status of parents according to the Canadian occupational classification (Canada, 2001).

**Table 1:** Comparison of means and standard deviations (parentheses) of clinical and demographic characteristics.

	Patients		Controls		*T*-test
	Mean	(SD)	Mean	(SD)	*t*
Age (years)	33	(7)	29	(9)	ns
SES parents	3	(1)	2	(1)	ns
WAIS block	8	(5)	12	(3)	^*^
WAIS vocabulary	7	(3)	10	(5)	ns
Laterality right (%)	79	–	81	–	ns
Luteal phase (%)	37	–	50	–	ns
Symptoms evolution (years)	9	(8)	–	–	–
CPz (equivalence in mg)	562	(325)	–	–	–
PANSS positive	16,5	(7)	–	–	–
PANSS negative	17	(8)	–	–	–
*PANSS general *	33,5	(11)	–	–	–

* *p* < 0.05. WAIS, Weschler Adult Intelligence Scale; PANSS, Positive and Negative Syndrome Scale, SES, socioeconomic status; ns, non significant; CPz, Chlorpromazine.

Experienced psychiatrists assessed all patients before assignation to the research group (DSM-IV-TR, criteria A to E); the schizoaffective and schizophreniform patients were excluded. Participants in the control group received a clinical screening interview with the SCID (First et al., 1996). The severity of symptoms was assessed with the positive and negative scale syndrome (PANSS; Kay et al., 1987). The onset of illness was defined as the date of the first psychiatric consultation. All patients had received at least an atypical neuroleptic (10 patients received a single type of neuroleptic and 8 received two; Clozapine: *n* = 9, average dose = 377.78 ± 90.11 mg; Olanzapine: *n* = 4, average dose = 15 ± 7.07 mg, Risperidone: *n* = 8, average dose = 4 ± 2.08 mg; Quetiapine: *n* = 5, average dose = 375 ± 309.23 mg). All doses of neuroleptics have been calculated according to the chlorpromazine equivalence (Woods, 2003). Exclusion criteria included an age below 18 or more than 45 years old, a past or present condition of neurological disorder or psychiatric axis-I disorder (other than schizophrenia in the group of patients), alcoholism or drug addiction, abnormal non corrected vision. The use of oral or injectable contraceptives in women and failure to follow test procedures and a non-corrected vision were also excluded. Those who were unable to give a blood sample at the day of EEG recording were systematically excluded. In total, 12 participants had to be excluded (6 men including 3 patients and 6 women including 4 patients). Three of the women were taking oral contraceptives, three of them have presented technical problems in the acquisition of EEG, two men had too many eye movements during EEG signal acquisition, and finally, four other were unable to give blood sample on the day of EEG recording.

A 10 ml blood sample was taken about 30 min before each EEG/ERP session to assess levels of gonadal hormones in all participants. Mean plasma levels are presented in **Table 2** for all groups. The sample was immediately centrifuged and the serum was separated. The samples have been preserved (at -40°C) and later transported and analyzed at the laboratory of the Maisonneuve-Rosemont Hospital (Montréal, QC, Canada). Hormone levels were determined using the automated determination by chemoluminescence (SYNCHRON LX^®^ i 725, Beckman Coulter, USA). For the testosterone, the analytical sensitivity was 0.1 ng/mL and the dynamic field of 0.1–9.9 ng/mL. For estradiol and progesterone, the analytical sensitivity was 20 pg/ml (73 pmol/L) and the dynamic field of 20–4800 pg/ml (73–17621 pmol/L).

**Table 2:** Mean plasma level of gonadal hormones and standard deviations (in parentheses) for each group.

	Women			Men	
	Patients (*n* = 9)	Controls (*n* = 13)	Mean	Patients (*n* = 9)	Controls (*n* = 11)	Mean
Progesterone	14 (13)	18 (22)	16 (17)	2 (2)	2 (1)	2 (1)
Estradiol	307 (132)	338 (278)	323 (205)	54 (32)	68 (24)	61 (27)
Testosterone	2 (1)	2 (1)	2 (1)	10 (5)	16 (5)	13 (5)

*The independent group *t*-tests were applied between patients and controls across both men and women groups. ns, non significant.*

In accordance with the Declaration of Helsinki, written informed consent was obtained from all participants before the commencement of the study. The capacity of the schizophrenia patients to give informed consent was established using the guidelines of the Canadian Psychiatric Association (Arboleda-Florez, 1997). The study was approved by the local ethics committees of the Louis-H Lafontaine Hospital and by the *Regroupement Neuroimagerie Québec* – Joint Research Ethics Committee.

### EXPERIMENTAL SETTING

On arrival at the laboratory, participants read and signed an informed consent form. Women were asked to come during day 4–8 or day 16–26 of their menstrual cycle, depending on its length, to compare the two-phase hormonal differences throughout the cycle. EEG recordings were made in a dimly lit room where each participant was seated in an adjustable chair in front of the computer monitor. The recording room constituted a separate corner of another room in which the experimenters, amplifiers, and computers were located. The nylon electrode cap, electro-oculogram, and mastoid references were installed within 30 min. A 1-min resting baseline was recorded at the beginning of the experiment to facilitate laboratory adaptation.

### STIMULI SELECTION

The emotional materials were photographic images from the International Affective Picture Systems (Lang et al., 2008) a standardized collection of images gathered from a wide variety of emotional and semantic categories. A total of 100 photographic images were chosen and classified into four groups, according to their valence and arousal level. These picture selections were based on the IAPS normative ratings for male and female separately, in order to control for valence (high and low valence images with similar arousal levels based on non-significant *t*-test comparisons) and arousal (high and low arousal with comparable valence based on non-significant *t*-test comparisons)^1^. Participants received 100 trials in total (25 high valence/low arousal, 25 high valence/high, 25 low valence/high arousal, 25 low valence/low arousal). These image categories were presented in different random orders to counterbalance potential sequencing effects. In each emotional category, the images contained the same basic attributes (scenes including humans, animals, inanimate objects or landscapes) across category in order to preserve coherence. Participants were informed that a series of pictures would be presented and they should pay attention to each during all the time of presentation. To ensure the continuous attention of participants, we asked them to press the keyboard spacebar, when they saw a human being in the picture (see Glaser et al., 2012 for similar procedures). The number of human being pictures was equivalent across the four experimental conditions (mean range = 1.12 and 1.64). In order to control for a possible impact of motor responses on ERPs, separate correlations between ERP components (P200/N200/P300) and motor responses were done, but failed to show any significant result (*r* range for the P300 between 0.08 and 0.20; for the N200 between 0.09 and 0.23; for the P200 between 0.10 and 0.30). Following the recording of EEG, pictures were shown again to participants and they were asked to rate subjectively the valence and the activation based on the standard Manikin self-assessment (Bradley and Lang, 1994).

All images were presented one at a time on a 19″ flatscreen monitor (NEC Accusync LCD 92vx), set with a 60 Hz refresh rate (32 bits), for a fixed duration of 3000 ms, at a distance of 90 cm calculated from the nose to the center of the computer screen with a 5° angle. Each picture was in JPG format, presented at a resolution of 1280 × 960 pixels with a maximum of 16.7 million colors (24 bits per pixel). The inter-trial interval (ITI) was fixed at 1000 ms, during which a red and white checkerboard image appeared (IAPS #7182). This red and white checkerboard image informed the participant to fixate on a point between picture presentations and reduced the eye movements. This procedure also helped to reduce the after image effect, which occurred during presentation of a white blank background in our previous pilot studies.

### EEG RECORDINGS AND ERP EXTRACTION

The EEG was recorded from 18 tin electrodes mounted in an elastic nylon cap (Electro-Cap International Inc.) The scalp electrodes were placed according to the guidelines for standard electrode position by the American EEG Society (1994) at frontal (F1, F2, F3, F4, F5, and F6), central (C1, C2, C3, C4, C5, and C6), parietal (P1, P2, P3, P4, P5, and P6) locations. All electrodes were referenced to linked mastoids and their impedances were kept below 5 KΩ. The Electro-oculograms (EOG) was recorded using four 9-mm tin external bi-polar electrodes. For the horizontal EOG, electrodes were placed at the outer canthus of each eye and for the vertical EOG at infra and supra-orbital points at the left eye, aligned with the pupil looking straight. A bioelectric digital amplifier model DBP A-1 (Sensorium, Charlotte, VA, USA) amplified the EEG signals (gain = ±5000) with a band-pass between 0.01 and 30 Hz (notch filter = 60 Hz). The EEG was recorded continuously at a sampling rate of 500 Hz and averaged offline in a time-window beginning at 100 ms before and until 1000 ms after picture onset. All epochs with a voltage exceeding ±100 μV and clippings due to saturation or blocking of the amplifiers were eliminated automatically during the averaging procedure. All EEG related to reaction times greater than 2000 ms were excluded online. A threshold of 16 trials free of both errors (false alarms and misses) and artifacts were accepted in the averaging, which is comparable to the criteria used in similar ERP experiments (Palomba et al., 1997; Maratos et al., 2000; Windmann and Kutas, 2001; Dolcos and Cabeza, 2002) and according with current guidelines (Duncan et al., 2009). Three time windows have been defined for the P200 (150–250 ms), the N200 (250–400 ms), and the P300 (300–600 ms). Our experimental hypotheses were tested using the baseline to peak amplitudes of these ERPs previously proposed as sensitive to emotional and attentional processes (Schupp et al., 2003; Kissler et al., 2009; Stewart et al., 2010).

### STATISTICAL ANALYSIS

Hormonal levels were submitted to a repeated measures MANOVA (SPSS^®^-Win19) with HORMONES (three levels: progesterone, estradiol, testosterone) as within factor and SEX and GROUP (schizophrenia, controls) as the between group factors. Behavioral and ERP data were submitted to repeated measures MANCOVA with VALENCE (two levels; pleasant/unpleasant) and AROUSAL (two levels; high/low). The analysis of the ERP data contained two additional within-subject factors related to cortical regions of HEMISPHERE (two levels: left/right) and REGION (two levels: anterior/posterior). The anterior region was populated by F1, F3, F5, FC1, FC3, FT7 (left hemisphere), F2, F4, F6, FC2, FC4 FT8 (right hemisphere), while the posterior region was populated by P1, P3, P5, C1, C3, C5 (left hemisphere) and P2, P4, P6, C2, C4, C6 (right hemisphere). Estradiol and testosterone levels were entered in each analysis as separate covariates. ERP analyses were carried out separately on each of the three temporal windows (P200, N200, and P300) and where significant results were obtained, additional *post hoc* tests were computed for multiple comparisons. In all analyses the significance level was set at 5% (two-tailed) with Huyndt-Feldt correction where necessary. The effect size measures for analyses of variance was estimated by the partial eta square ($\eta_{p}^{2}$) for each significant interactions to estimate the degree of association between variables for the sample. Separate analyses were applied to assess the correlation (Pearson) between gonadal hormones (estradiol and testosterone) and the N200/P300 amplitude. Other correlations were applied between P300 amplitude and PANSS global scale and subscales in schizophrenic patients.

## RESULTS

### SUBJECTIVE EVALUATIONS OF EMOTIONAL IMAGES

Subjective assessments of emotional valence were consistent with the IAPS norms (see **Tables 3** and **4**). For each group, subjective assessments did not exceeded one standard deviation from the normative data. Two ANOVAS were conducted separately to compare ratings in the valence and arousal for controls versus schizophrenic groups and for men versus women. The ANOVA on the subjective evaluation of arousal has shown significant differences between high arousal and low-arousal, all groups combined [*F*(1.33) = 80.35, *p* < 0.001]. There is no significant difference between group and sexes in the subjective evaluation of arousal. The ANOVA on the subjective evaluation of valence has shown significant differences between high valence (pleasant) and low valence (unpleasant), all groups combined [*F*(1.33) = 190.27, *p* < 0.001]. There was no significant difference between groups in the subjective evaluation of valence.

**Table 3:** Comparison of the mean arousal and valence ratings between the International Affective Picture System MALE norms (from the Center for the Study of Emotion and Attention, Lang et al., 2008) and the current study subjective evaluations for the controls and our patients.

Category		IAPS Male normative data		Controls (*n*=10)		Patients (*n*=7)	
Valence	Arousal	Valence	Arousal	Valence	Arousal	Valence	Arousal
Pleasant	High	6,47	5,78	6,12	4,59	6,87	5,05
	Low	6,16	3,73	6,16	2,25	6,97	3,01
Unpleasant	High	3,25	5,86	3,38	5,54	3,36	5,99
	Low	3,62	4,11	3,81	3,95	4,54	4,55

**Table 4:** Comparison of the mean arousal and valence ratings between the International Affective Picture System FEMALE norms (from the Center for the Study of Emotion and Attention, Lang et al., 2008) and the current study subjective evaluations for the controls and our patients.

Category		IAPS Female normative data		Controls (*n*=11)		Patients (*n*=8)	
Valence	Arousal	Valence	Arousal	Valence	Arousal	Valence	Arousal
Pleasant	High	6,22	5,95	6,59	4,52	5,94	4,94
	Low	6,44	3,99	6,37	2,92	6,56	3,11
Unpleasant	High	3,21	6,14	2,83	5,92	2,96	6,18
	Low	3,49	4,22	3,83	4,48	4,24	4,11

### GROUP DIFFERENCES IN PLASMA GONADAL HORMONAL LEVEL

The hormone by sex was significant [*F*(2,37) = 16.27, *p* < 0.001; $\eta_{p}^{2}$ = 0.47; power = 0.99], which confirmed that men have higher testosterone level, while women have higher progesterone and estradiol level (see **Table 2**). However, there is no significant group by sex (*p* = 0.85), group by hormone (*p* = 0.74) nor any group by sex by hormone (*p* = 0.24), which underline that there is no significant sex differences within groups or between schizophrenia patients and controls across estradiol, progesterone or testosterone levels.

### CORRELATION ANALYSIS BETWEEN ERPs AND HORMONES

The correlation analysis between ERP and gonadal hormone status showed several significant results, except for low arousal unpleasant images. In the control group, the N200 amplitude was positively correlated with the estradiol level in all conditions, revealing that the N200 was reduced (less negative) when estradiol level was higher (*r* range from 0.37 to 0.49). Conversely, the N200 amplitude was not significantly correlated with the testosterone level (all *r*’s between -0.16 and -0.34). The anterior P300 amplitude to pleasant stimuli was positively correlated with estradiol level, revealing that the higher the level of estradiol, the larger the P300 amplitude (*r* ranged from 0.23 to 0.36). In contrast, the level of testosterone was negatively correlated with the P300 amplitude to pleasant stimuli (*r* range from -0.36 to -0.53), which means that with higher testosterone level, the P300 was reduced (**Table 5**). Importantly, all correlations between gonadic hormones and ERPs (N200 and P300) failed to reach significance, in response to unpleasant images (all *r*’s between 0.16 and 0.30). In the patient group, despite a tendency comparable to the control group, no correlation between ERP and hormone levels reached significance (**Table 6**).

**Table 5:** Correlations between gonadal hormones and N200/P300 amplitude in response to arousal and valence conditions across regions (control group).

Arousal	Valence	Regions	Estradiol		Testosterone	
			N200	P300	N200	P300
High	Pleasant	Anterior	0,45^*^	**0,38**	**-**0,26	-0,36^*^
		Posterior	0,37^*^	0,36	**-**0,18	-0,41^*^
	Unpleasant	Anterior	0,47^*^	0,30	**-**0,17	**-**0,18
		Posterior	0,48^*^	0,33	**-**0,24	**-**0,30
Low	Pleasant	Anterior	0,49^*^	0,46^*^	**-**0,34	-0,52^**^
		Posterior	0,47^**^	0,37^*^	**-**0,28	-0,53^**^
	Unpleasant	Anterior	0,27	0,33	**-**0,17	**-**0,30
		Posterior	0,20	0,16	**-**0,16	**-**0,28

* *p*< 0.01; **p < 0.001.

**Table 6:** Correlations between gonadal hormones and N200/P300 amplitude in response to arousal and valence conditions across regions (Schizophrenia group).

Arousal	Valence	Regions	Estradiol		Testosterone	
			N200	P300	N200	P300
High	Pleasant	Anterior	0,19	0,27	**-**0,18	**-**0,27
		Posterior	0,34	0,20	**-**0,05	**-**0,24
	Unpleasant	Anterior	0,32	0,34	**-**0,19	**-**0,26
		Posterior	0,40	0,26	**-**0,22	**-**0,37
Low	Pleasant	Anterior	0,08	0,36	**-**0,07	**-**0,24
		Posterior	0,34	0,28	**-**0,23	**-**0,34
	Unpleasant	Anterior	0,26	0,25	**-**0,26	**-**0,23
		Posterior	0,39	0,36	**-**0,33	**-**0,30

### EVENT-RELATED POTENTIALS^2^

#### P200 component

Overall, the amplitude of the P200 component was 2.7 μV (SE = 4.38) with an average latency of 200 ms (SE = 30.82). The scalp distribution was more prominent in the posterior region [*F*(1.38) = 13.99, *p* < 0.05] and there was no main effect or interaction across groups.

#### N200 component

Overall, the amplitude of the N200 component was -6.58 μV (SE = 5.66) and was more prominent over the anterior than over the posterior regions [*F*(1,40) = 115.17, *p* < 0.001; $\eta_{p}^{2}$ = 0.74; power = 0.99]. The N200 peaked at an average latency of 320 ms (SE = 23.10). A valence by arousal by region interaction was significant [*F*(1.39) = 7.74, *p* < 0.005] only after covarying for testosterone, showing a larger valence effect for high arousal than low arousal images over frontal regions. This interaction was not significantly different between groups. A group × hemisphere interaction [*F*(1,40) = 4.89, *p* < 0.05; $\eta_{p}^{2}$ = 0.11; power = 0.59] was significant and remain present after covarying for estradiol [*F*(1,39) = 4.85, *p* < 0.05; $\eta_{p}^{2}$ = 0.11; power = 0.57] and testosterone [*F*(1,39) = 6.12, *p* < 0.05; $\eta_{p}^{2}$ = 0.14; power = 0.68]. This hemispheric asymmetry is expressed by a significantly larger negative amplitude for the right (-7.43 μV) than for the left hemisphere (-6.41 μV), among the control group [*F*(1,23) = 5.73, *p* < 0.05], while patients with schizophrenia did not demonstrate this hemispheric asymmetry [*F*(1,17) = 0.29, *p* > 0.05]. All possible main effects or interaction with emotional valence and arousal factors were not significant across all regions at that latency.

#### P300 component

Overall, the mean amplitude of the P300 component was 2.66 μV (SE = 0.77) with mean latency of 370 ms (SE = 71.13) and a scalp topography, mainly predominant in the posterior region [*F*(1.40) = 47.98, *p* < 0.001]. A main effect of valence [*F*(1.40) = 9.25, *p* < 0.005] and a valence by arousal interaction [*F*(1.40) = 16.70, *p* < 0.001] was present revealing a larger valence effect in high arousal stimuli. A main effect of hemisphere [*F*(1.40) = 9.10, *p* < 0.005; $\eta_{p}^{2}$ = 0.19; power = 0.84] and a group × hemisphere [*F*(1.40) = 8.12, *p* < 0.01; $\eta_{p}^{2}$ = 0.17; power = 0.80] was present and remained significant after covarying for estradiol [*F*(1.37) = 9.91, *p* < 0.005; $\eta_{p}^{2}$ = 0.21; power = 0.87] and testosterone [*F*(1.37) = 5.83, *p* < 0.05 η = 0.14; power = 0.65] levels. Generally, the P300 amplitude was larger over the right hemisphere in the schizophrenic group, which was not the case in controls.

**FIGURE 1:**
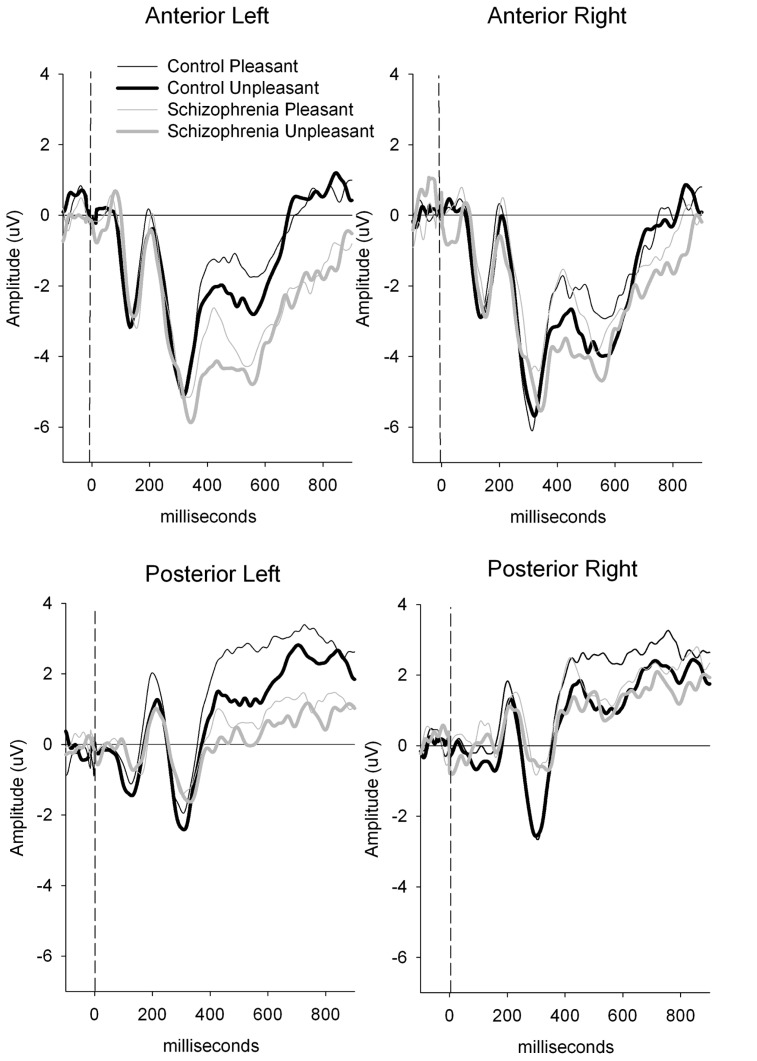
**Clinical group comparison between anterior and posterior P300 amplitude.** P300 amplitude is generally smaller in schizophrenic patients compare to the control group in all conditions, but differences between pleasant and unpleasant are larger over anterior region in this group.

**FIGURE 2:**
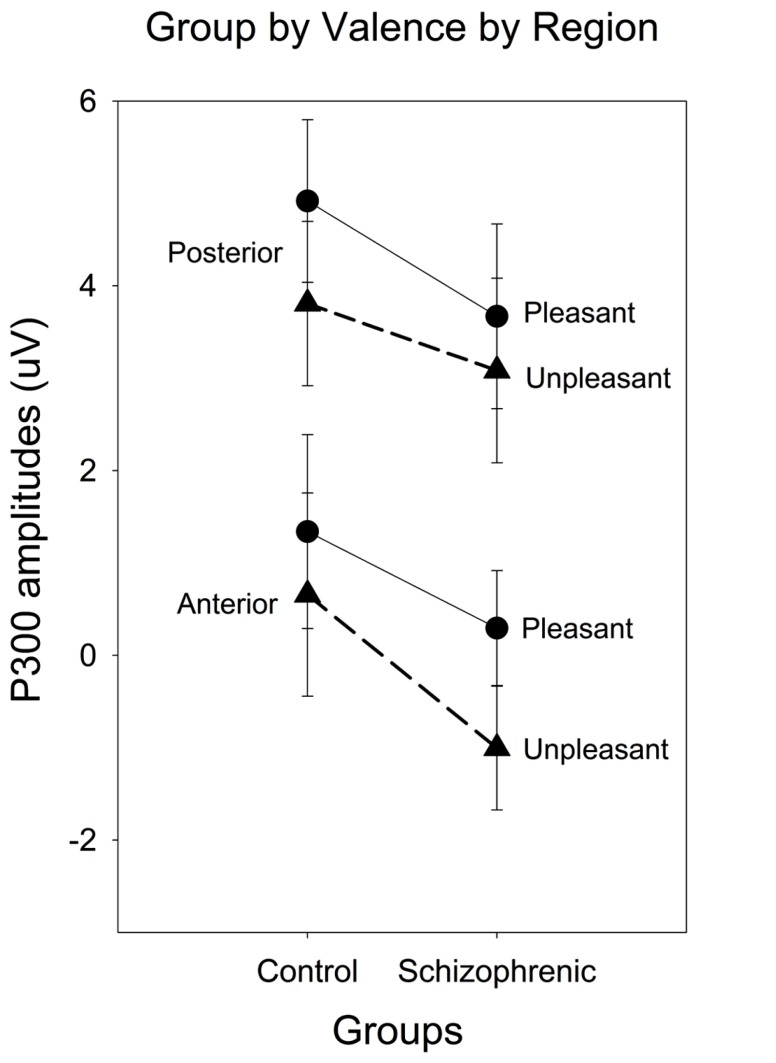
**Event-Related Potential (ERP) waveforms for anterior and posterior regions and for left and right hemisphere comparing schizophrenia patients (gray lines) and controls (black lines), contrasting pleasant (solid thin lines) and unpleasant (bold thick lines)**.

A group × valence × region interaction [*F*(1.40) = 5.24, *p* < 0.05; $\eta_{p}^{2}$ = 0.12; power = 0.61] was present and remained significant after covarying for estradiol [*F*(1.39) = 5.46, *p* < 0.05; $\eta_{p}^{2}$ = 0.12; power = 0.63] and testosterone [*F*(1.39) = 4.55, *p* < 0.05; $\eta_{p}^{2}$ = 0.11; power = 0.55]. To decompose this interaction, a subsidiary analysis done separately for the control and schizophrenic group, indicated that the valence by region interaction was only significant in the patient group [*F*(1.16) = 4.81, *p* < 0.05; $\eta_{p}^{2}$ = 0.23; power = 0.55]. In schizophrenic groups, a significantly smaller amplitude was observed to the unpleasant compared to the pleasant {valence effect [*F*(1.17) = 4.94, *p* < 0.05; $\eta_{p}^{2}$ = 0.23; power = 0.55} and this valence effect was larger over the anterior region {region effect [*F*(1.17) = 31.33, *p* < 0.001; $\eta_{p}^{2}$ = 0.65; power = 0.99} (see **Figures 1** and **2**). There was also a negative correlation between the anterior P300 amplitude related to unpleasant valence and the negative (*r* = 0.51, *p* < 0.05) symptom scale of the PANSS. In other words, higher negative symptoms are correlated with smaller frontal amplitude in response to unpleasant images.

## DISCUSSION

In the present work, we first examined the temporal signature of emotional processing on ERP activations, to compare individuals with schizophrenia and matched controls. Also, we have considered the impact of schizophrenia and gonadal hormone levels, while taking into account the separate contributions of emotional valence and arousal processing. First, our behavioral results showed that patients with schizophrenia were able to subjectively assess valence and arousal images comparably to control participants. In addition, emotional valence or arousal failed to affect early P200 and N200 components, which reflect early processes of selective attention, whereas emotional valence influences the later P300 component, which is related to memory context updating, and stimulus categorization. At that latency range, specific group differences were observed for the anterior P300 related to the unpleasant image presentations. We also proposed the hypothesis that the insertion of gonadal hormones as covariates should reveal a certain modulating impact on electrocortical activity. Even if no differences were found across groups, in gonadal hormones, we found correlations between gonadal hormone levels and ERP brain responses, specifically under unpleasant emotions. Let’s now decompose these effects according to specific components.

### P200 AND N200 EFFECT OF EMOTION AND DIFFERENCES IN SCHIZOPHRENIA

We initially hypothesized that, individuals with schizophrenia process emotional material differently than matched controls. First, emotional processing related to schizophrenia did not appear to influence early cognitive processes, indexed by the P200 component. Regarding the functional interpretation of the P200, some analyses using dipole sources showed that this component could be associated with a frontal and medio-ventral generator, involving especially the orbitofrontal and the anterior cingulate cortex (Potts et al., 1996, 2004; Potts, 2004). These cortical regions would be related, in part, to motivation and preliminary analysis of the stimulus. In the context of psychosis, the current study provides data on the emotional temporal dynamic, by showing that emotion processing, at the P200 level, seems relatively intact in patients with schizophrenia. A study of Horan et al. (2010), carried out with similar images from the IAPS, also showed P200 amplitude quite comparable to matched controls. The response of the P200 should thus, reflect early stimuli discrimination. The increase of the electrocortical activity, at that latency, represents a relatively automatic attention process (Ferreira-Santos et al., 2012), suggesting that early cognitive and emotional processing stages are relatively intact in individuals suffering from schizophrenia. In contrast, findings of Pinheiro et al. (2013) suggest that visual evocative stimuli are differentially processed within the first 200 ms, and that the early stages of visual evocative stimuli processing are abnormal in schizophrenic patients, irrespective of stimulus valence. However, they proposed a blocked design protocol with different selections of IAPS images, while we used a parametric design comparing 2 (high–low) valence by 2 (high–low activation) images, which could explain, in part, the discrepancies. In addition, they also selected only male participants that were more symptomatic (PANSS = 41) than the ones in our sample (PANSS = 33). So, it’s possible that early ERP differences appear when a certain threshold of symptoms is crossed. Further analyses are much needed with larger samples, including a wider array of symptoms intensity and a comparison between males and females.

Later in the processing stream, we also found notable differences in the N200 component, susceptible to confirm electrocortical sensitivity to emotional stimuli in schizophrenia. Our results showed a general right hemisphere lateralization of the N200 in the control group, which was not present in the schizophrenic group. In a literature review focusing on right hemisphere functions in schizophrenia, Mitchell and Crow (2005) pinpointed some evidence indicating that patients with schizophrenia and patients with right hemisphere lesions share common deficits in understanding emotional prosody, including humor, sarcasm, and metaphors. According to Kucharska-Pietura (2006), the poorer performance of emotional tasks by right-hemisphere-damaged patients compared to other groups might support right-hemisphere superiority for affective perception. Consistent with that hypothesis, Ribolsi et al. (2009) have reported reduced brain lateralization in schizophrenia, causing a failure of left hemisphere dominance. Based on prior findings, the amplitude of the N200 is related with the preliminary evaluation of image characteristics, guided by the activation of perceptual qualities of images (ex: color, context, etc.) for a subsequent higher level of processing (Dolcos and Cabeza, 2002; Schupp et al., 2004). Specifically, this component could underline the contribution of the anterior cingulate cortex and the medial prefrontal cortex in the evaluation and expression of an unpleasant valence emotion (Viinikainen et al., 2010). In addition, these two regions have a role to play in regulating limbic regions, involved in the generation of emotional responses (Etkin et al., 2011).

### P300 EFFECT OF EMOTION IN SCHIZOPHRENIA

Later, there is the P300 component that showed interesting results, allowing contrasting specific differences in schizophrenia, in relation with emotional valence. This P300 component is typically associated with attention and memory context updating (Hajcak et al., 2010). If we transpose that to the context of our clinical population, individuals suffering from schizophrenia often showed a deterioration of endogenous mechanisms related to attentional control, which would be a direct consequence of the reduced P300 amplitude. This finding also fits well within the model proposed by Dolcos and Cabeza (2002), which postulate that the posterior brain region respond to emotional images. These stimuli may modulate attention resources and prioritize some information because of the intrinsic importance of this type of emotional stimuli. Other fMRI studies have consistently revealed an increase in neuronal activity in the occipital and parietal cortex, as well as in the lower temporal cortex in response to high arousal images (Bradley et al., 2003; Sabatinelli et al., 2005; Junghofer et al., 2006). These regions would later reflect the effect of explicit attention to target images (Schupp et al., 2007).

Moreover, our P300 group differences were relatively specific to the valence dimension, with amplitude response to unpleasant stimuli, correlated with the anterior region. Past investigations showed that unpleasant stimuli seem to evoke automatic and peripheral physiological responses, more easily than pleasant emotional ones (Gross, 2002; Ohira et al., 2006), which is consistent with the orbitofrontal, the superior frontal gyrus, anterior cingulate cortex and amygdala involvement in the processing of unpleasant emotions (Mak et al., 2009; Rauch et al., 2010). This could explain, in part, the frontal P300 depletion, in response to unpleasant stimuli. The orbito-frontal gyrus would be associated with the voluntary inhibition of unpleasant emotions, as well as a top-down regulation of automatic and peripheral responses to emotional experience (Phillips et al., 2003; Ohira et al., 2006). Furthermore, some studies have also suggested that the orbito-frontal region participate actively to learning reversal (Fellows and Farah, 2003; Hornak et al., 2004; Kringelbach and Rolls, 2004), as well as the perception of an emotional event (Hynes et al., 2006). These considerations might suggest that the regulation of emotions is accomplished by updating the emotional context of a stimulus (Ochsner et al., 2002), which would explain the involvement of the P300 when processing unpleasant stimuli.

### EMOTION AND GONADAL HORMONE DIFFERENCES IN SCHIZOPHRENIA

In addition, the analyses of gonadal steroid hormones allowed assessing whether these hormones intervene in modulating cerebral activity. In favor of the insertion of these factors, animal, and human investigations indicates that sex steroids can have direct effects on the development of certain psychopathologies (Alexander and Peterson, 2001) and as a result, some gonadal and adrenal hormones could play a significant role, hitherto underestimated, in the pathophysiology and the evolution of schizophrenia. Seeman and Lang (1990) hypothesized that estrogens, modify symptom expression and account for many of the observed sex differences, while Hafner et al. (1993), suggested that estradiol may play a protective role in schizophrenia at least until the menopause. Others suggested that sex differences should be rather attributed to testosterone, which may play a role in the etiology of schizophrenia (Salokangas, 1995). Indeed, testosterone levels increase significantly, reaching a peak during adolescence in men, and then gradually decline with age (Wilson, 1996), which follows approximately the pattern of schizophrenia onset. However, other studies indicated a delayed puberty and low testosterone in men suffering from schizophrenia (Kline et al., 1968). More recent studies have shown that plasma levels of testosterone were inversely proportional to the severity of negative symptoms in male with schizophrenia (Shirayama et al., 2002; Akhondzadeh et al., 2006; Ko et al., 2007). A study carried out in our laboratory, found a decrease in testosterone levels in male control patients, compared to the controls of the same sex groups and, surprisingly, increased levels have been observed in patients (Mendrek et al., 2011). In addition, the high levels of testosterone in women were correlated with brain activations during a mental rotation task (Mendrek et al., 2011). For the estrogen in general, symptoms seem to improve when estradiol levels rise, and vice versa. This can be interpreted as a probable evidence for a protective effect of estrogens in schizophrenia, possibly due to the anti-dopaminergic activities of estrogens (Riecher-Rossler et al., 1994). Estrogen also stimulates a significant increase of 5-HT2A in frontal, cingulate, olfactory cortex and in the nucleus accumbens (Fink et al., 1996). This is consistent with activation of cerebral areas related to the control of mood, cognition, emotion in schizophrenia. However, our results showed that, even when taking into account these hormonal levels, our interactions remained significant before and after the insertion of these covariates. But the absence of impact of these covariates may be due to our small sample, because the results showed that there is indeed a correlation between ERPs and gonadic hormones and between psychosis intensity and cerebral activity.

These results allow us to hypothesize that a change in hormonal levels has a mediating impact on electrophysiological measures, at least, in the context of an emotional protocol. This approach remains important and original in our experimental context, because hormonal variations within a gender group can also be important and alter cerebral activity. In support of our findings, we need to underline that gonadal hormones of our samples were relatively comparable between patients and controls, except for estradiol level that was slightly reduced in schizophrenic women, a finding also observed in other investigations (Riecher-Rossler et al., 1994; Kulkarni et al., 2011). Our results showed that the anterior P300 amplitude to pleasant stimuli was positively correlated with estradiol level. These results are comparable to earlier findings from Krug et al. (2000), with the presentation of emotional stimuli at different stage of menstrual cycle in healthy women. During the ovulatory phase, amplitude of a late positive component (similar to our P300) was larger to sexual stimuli than that evoked by the neutral or unpleasant images. These data indicate a specific effect of estradiol variations on the processing of pleasant stimuli.

Moreover, our results also showed that the level of testosterone was negatively correlated with the P300 amplitude to pleasant stimuli. There is no comparable result in the literature, but with healthy participants endogenous testosterone concentrations are generally positively correlated with amygdala and orbito-frontal responses and testosterone increases amygdala reactivity (van Wingen et al., 2011). On the other hand, with schizophrenia patients, a significant inverse correlation was noted between negative psychosis symptoms and plasma levels of testosterone in patients with predominant negative symptoms (Akhondzadeh et al., 2006), which is partly consistent with our finding of higher negative symptoms that correlate with smaller frontal P300 amplitude in response to unpleasant images. From our results and from the literature altogether, we can propose that a lower level of testosterone could be related to negative symptoms, which in turn favor depleted anterior P300 amplitude in response to unpleasant stimuli.

### LIMITATIONS

With the current study, several potential limitations need further considerations. First, we agree that group sampling should include a greater number of participants, both to represent further the general population and the clinical group, recruiting as many women as men for each group. Another limitation lies in the level of functioning, since the patients in our study were clinically stable and relatively well-functioning. Future studies will have to establish to what extent the current results can be generalized for all individuals affected by schizophrenia. In addition, it would be interesting to look at the potential implications of antipsychotic drugs on the results obtained in patients, since they all took antipsychotic medications, which varied individually in dosage and titration. The effects of different molecules of antipsychotic drugs on the emotional experience are not very clear, although evidence suggests that such effects are minor (Berenbaum and Oltmanns, 1992; Kring and Neale, 1996). However, more recently, several studies were able to demonstrate interactions between certain types of antipsychotic medication and cognitive functions (Davidson et al., 2009; Marston et al., 2009; Mucci et al., 2011), a situation which could produce effects on emotions. Given that we are investigating hormonal differences, it would be more relevant to divide each group of women, according to either luteal or follicular phase of the menstrual cycle at the time of EEG/ERP recording.

## CONCLUSION

In sum, the current data showed specific differences in patients with schizophrenia on several temporally and spatially distinct ERP components coupled with emotional effects. Furthermore, our results place into perspective the involvement of specific emotional dimensions on a continuous temporal stream. Thus, emotional pleasantness (i.e., valence) would impact more on the anterior N200 components associated with selective attention and evaluation of primary characteristics of the stimuli, as well as the dimension of arousal. The arousal would be, therefore, processed later and thus associated with subsequent higher cognitive processing, as reflected by the P300 component (Dolcos and Cabeza, 2002; Glaser et al., 2012). The next logical step was to examine the potential involvement of gonadal hormones in these differences; because they are known to act on the regulation of emotional responses and represent a basic and measurable physiological difference between men and women. These data clearly demonstrate that endocrinological variables could help monitor the relationship between emotion processing, electrocortical activity and the development of symptoms and that we need to deal with this reality in future research. The functional significance of these results remains to be determined in a comprehensive psychoneuroendocrinological study.

## Conflict of Interest Statement

The authors declare that the research was conducted in the absence of any commercial or financial relationships that could be construed as a potential conflict of interest.

## Footnotes

1 In bold: stimuli presented to women only; underlined: stimuli presented to men only; other stimuli were presented to both sexes. *Pleasant valence - high arousal:* 1101 **1303** 1321 2025 2160 2389 4004 4230 **4533 4561** 4601 **4607 4608** 4609 4623 4641 **4651 4652** 4653 **4664 4666 4672 4676 **4661 4810 5450 5623 **5626** 5920 7570 7600 8031 8120 8130 8160 **8192 **8200 **8251 8400** 8475 *Pleasant valence - low arousal* : 1500 1610 **1661** 1910 2005 2010 2070 2092 2222 **2370 **2394 **2442 **2510 2515 **2600 **2660 2745.1 2749 **4536** 4614 4625 5201 5760 5849 **7039 7205 **7207 7285 **7286 **7340 **7493 7500 7503 7820** 8032 8220 8460 Unpleasant valence - high arousal: 1040 1070 **1080 1101 1113 1114 **1200 1220 **1300 1930 1931** 2352 2661 2683 **3022** 3150 3160 **6020** 6200 **6211** 6242 6250 6315 6370 6530 **6555** 6560 **6836 6840** 7359 **8480** 8485 9160 **9230** 9250 9270 **9373 9480** 9490 9600 **9621 9630** 9910 9921 Unpleasant valence - low arousal: **1230** 2055” 2095 2205 2278 **2280** 2399 **2441 **2520 2695 2700 2752 2810 **2900.1** 3300 **4003 4279 4290** 4490 4530 4572 6561 **7700 **9000 9041 **9045 **9110 **9171 **9190 9265 **9331 **9341 **9360** 9417 9452 9471 9584 **9912**

2 Because certain correlations between ERPs and gonadal hormones were significant, we inserted the testosterone and estradiol as covariates in the MANOVA for the N200 and the P300 separately. If we keep separate men and women, the results showed no correlation between estradiol and testosterone (*r* = 0.19) in women, while men showed a significant correlation between these two hormones (*r* = 0.63). For that reason, we chose to keep separate, the insertion of covariance in our ANOVAs (i.e., the repeated measures ANOVA were done with estradiol, then the testosterone separately).
